# The Music-Related Quality of Life Measure (MuRQoL): A Scoping Review of Its Validation and Application

**DOI:** 10.3390/audiolres15020026

**Published:** 2025-03-07

**Authors:** Giorgos Dritsakis, Andrea Frosolini, Cynthia Lam

**Affiliations:** 1 Institute of Communication and Computer Systems, National Technical University of Athens, 15773 Athens, Greece; 2 Department of Maxillofacial Surgery, Policlinico Le Scotte, University of Siena, 53100 Siena, Italy; andreafrosolini@gmail.com; 3 SOUND Lab, Cambridge Hearing Group, Department of Clinical Neurosciences, University of Cambridge, Cambridge CB2 3EB, UK; cccl4@cam.ac.uk

**Keywords:** music, quality of life, perception, engagement, cochlear implant, importance, translation, validation, rehabilitation, questionnaire, PROM, PICO

## Abstract

Background: The Music-Related Quality of Life (MuRQoL) was launched in 2017 as a valid psychometric measure of Cochlear Implant (CI) users’ music experience and its impact on Quality of Life (QoL). This scoping review aimed to explore the implementation and effectiveness of the instrument since its introduction. Methods: PubMed and Google Scholar databases were searched for publications written in English reporting a translation, validation or application of the MuRQoL. Results: Eleven publications were found, including three validated versions and eight studies that applied the MuRQoL in Italy, Turkey, Spain and the US in research and clinical settings for various purposes. The review showcases the reliability, validity and potential clinical utility of the tool underscoring its growing adoption and integration with other assessment tools. Based on the findings, we make recommendations and provide a roadmap for optimizing the use of MuRQoL globally, including naming and terminology conventions. Anecdotal evidence, such as forthcoming or non-English publications offer further insights into the tool’s future potential. Conclusions: The MuRQoL is currently available in four languages and applicable across diverse cultural contexts, populations and settings. It is a versatile, patient-centered tool providing a deep understanding of CI users’ music experiences. As research and validation efforts continue, the instrument has the potential to set a benchmark for assessing the broader impacts of CIs beyond speech perception, emphasizing the significance of music in enriching the lives of individuals with Hearing Loss (HL).

## 1. Introduction

Music is a universal and enriching element of human life, playing an important role in emotional expression, social connection and personal well-being. While listening to music may seem effortless, it is among the most complex auditory and cognitive tasks, requiring the intricate processing of pitch, timbre, rhythm and harmony [1]. This complexity makes music listening particularly challenging for individuals with hearing loss (HL), especially cochlear implant (CI) users. CIs have revolutionized auditory rehabilitation for people with severe-to-profound HL as they offer substantial improvements in speech understanding (especially in favorable listening conditions), communication and quality of life (QoL), particularly in the first year of CI use [2]. However, as CIs are designed primarily for speech, coarse spectral resolution and limited transmission of spectro-temporal fine structure impede accurate perception of fundamental musical elements such as pitch, melody and timbre. While central neural adaptation with CI use partially mitigates these limitations, they often still result in diminished music perception and, consequently, reduced emotional engagement and enjoyment, which may negatively affect QoL [3,4,5]. Despite the poor perception of fine acoustic features, music remains meaningful to many CI users, providing a source of enjoyment, identity, and emotional connection. Research shows that music engagement in CI users is influenced by many other factors beyond CI technology, including pre-implant musical exposure, personal expectations and adaptive strategies [6,7,8]. Understanding how CI users experience music is critical for improving their relationship with music [9,10].

In recent years, the scope of CI research has expanded from a traditional emphasis on speech perception to a broader consideration of music perception and enjoyment. Key advancements in the field include the stronger consideration of patient opinions and experiences in shaping and delivering research projects [11]. There is also a better understanding of the specific musical cues, such as rhythm and tempo, which are more accessible to CI users. These insights have informed the design of innovative signal-processing strategies aimed at enhancing those cues, such as algorithms that emphasize harmonic structures or optimize dynamic range [12]. Additionally, tailored music training programs have been effective in empowering CI users to engage with music more confidently [13]. These programs often incorporate structured auditory exercises, real-world music exposure, and counseling to manage expectations. Collectively, these advancements represent a paradigm shift in auditory research and rehabilitation, moving from narrow technical improvements to a more holistic, patient-centered approach that values the broader context of music enjoyment and its emotional and social dimensions [14].

This transition toward a holistic perspective requires reliable and comprehensive methods to assess not only the technical aspects of music perception, but also the subjective, personal significance of music in the lives of CI users. Traditional research has largely focused on measurable acoustic elements, such as pitch accuracy, rhythm discrimination, and melody recognition, which, while valuable, do not capture the broader experience of music as a source of joy and connection [15]. There is now a growing recognition of the need to evaluate music’s impact on QoL, addressing questions of how music influences emotional health, social participation, and personal identity for CI users. Patient-reported outcome measures (PROMs) are essential in this context, as they provide a structured way to capture CI users’ lived experiences, preferences and goals. They facilitate the alignment of research and clinical interventions with what matters most to patients, thus allowing us to evaluate the true effectiveness of rehabilitation strategies [16]. In the context of music and CIs in particular, the absence of a standardized, widely accepted PROM has resulted in inconsistent research methodologies and a limited capacity to measure the success of interventions systematically [17]. This lack of a standard measure along with the advances in CI technology and rehabilitation techniques highlight the urgent need for reliable tools that not only evaluate outcomes but also guide individualized approaches to music rehabilitation [18].

To address the critical need for assessing the music experiences of CI users, the Music-Related Quality of Life (MuRQoL) questionnaire was introduced in 2017. Although the instrument was designed primarily as a tool to guide music rehabilitation in adult CI users, it had the ambition to become a standardized instrument for assessing the music-related QoL of CI users comprehensively [19]. Among its advantages over other music questionnaires for adult CI users (e.g., Munich Music Questionnaire, MuMu, Iowa Musical Background Questionnaire, IMBQ, University of Canterbury Music Listening Questionnaire, UCMLQ) are its psychometric properties, uniform response scale and assessment of importance of music and, subsequently, its impact on QoL. Since its introduction, both the questionnaire and its accompanying instruction sheet have been freely accessible through various platforms, including the original publication, the University of Southampton repository and direct communication with the authors. While a review article has reported the MuRQoL as particularly notable for its specific focus on guiding music rehabilitation, at the time of the review publication, there were no documented practical applications beyond its initial validation and psychometric evaluation [17]. Over the past few years though, there has been an increasing interest in the translation and adaptation of the instrument into multiple languages and diverse cultural contexts, as well as its utilization in a wide range of research and clinical settings. Journal publications, dissertations, requests for permission to use and other expressions of interest reflect the growing recognition of the MuRQoL as a valuable tool for both research and clinical practice in the field of music and CIs.

This review aims to explore the application, adoption, validity and effectiveness of the MuRQoL questionnaire in various settings since its introduction in 2017. Following from these, the review will assess its potential future role in CI users’ music rehabilitation and identify opportunities for future work.

## 2. Materials and Methods

**Figure 1:**
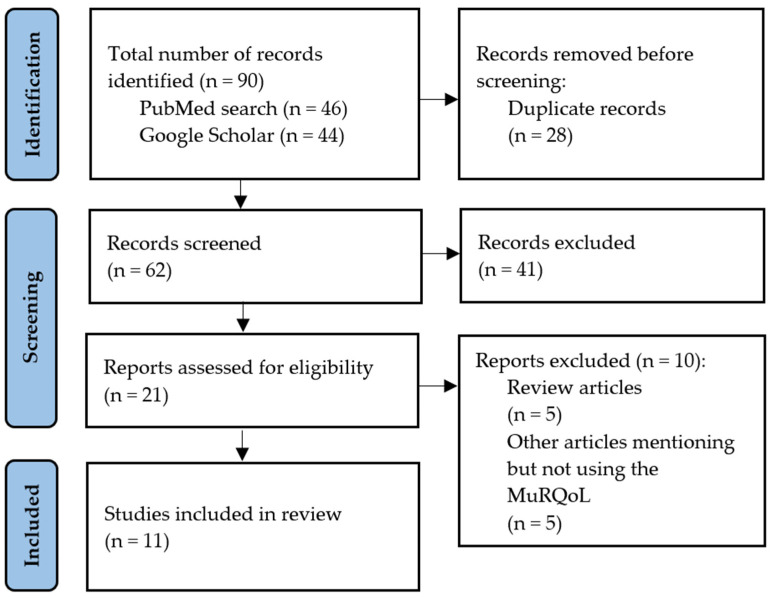
PRISMA (Preferred Reporting Items for Systematic Reviews and Meta-Analyses Extension for Scoping Reviews) flow diagram of the stages of the scoping review.

A scoping review was considered the most appropriate study design for the purpose of the present manuscript. Scoping reviews are commonly used in the literature to explore the use of previously developed questionnaires [20]. We conducted our review according to the Joanna Briggs Institute guidelines to ensure quality and reliability [21]. We followed the Preferred Reporting Items for Systematic reviews and Meta-Analyses (PRISMA) 2020 guidelines as well as the specific guidance for scoping reviews [22,23]. The completed PRISMA flow diagram can be found in Figure 1 and the full PRISMA checklist can be found in the Supplementary Material of this article. We searched the PubMed database using two keywords: ‘MuRQoL’ and ‘Music-Related Quality of Life’. We searched for publications written in English from 2017, when the original validation article was published, to date. Additionally, we searched PubMed and Google Scholar for articles that cited the original manuscript publication [19]. Google Scholar allowed us to expand the search to other material beyond journal articles. The search was repeated before submission to ensure that any newly published articles were included. Title and abstract screening and full text review were performed according to predefined eligibility criteria. We included original research publications that reported a translation, validation or application of the MuRQoL in a non-English language. Review articles mentioning the instrument without using it were excluded. Article identification and screening was performed by the first author and reviewed by the last author.

From all the studies included in the review we extracted data related to the administration and scoring of the MuRQoL, the population studied, the main findings, reliability and validity, results and conclusions. We performed a narrative, descriptive data synthesis in order to collectively report and discuss the purpose of MuRQoL use, administration and scoring, psychometric properties, the population in which the questionnaire was applied and the main findings. Results were organized and presented according to the PICOS framework, which allowed for a systematic and comprehensive synthesis of findings [24].

The original MuRQoL questionnaire can be found in the Supplementary Material of this article and is also summarized in Table 1. Part I assesses the perception of various music elements, music enjoyment and engagement in musical activities. Part II assesses the importance of each of those experiences.

Each questionnaire item is scored using a uniform 5-point Likert scale (one for ‘Frequency’ and one for ‘Importance’) that can also be transformed to a 0–100 score for each subscale. The original validation study demonstrated high internal consistency, test–retest reliability, and construct validity, with statistically significant differences between CI users and NH individuals, as well as correlations with QoL measures [18]. The MuRQoL was designed to serve a dual purpose: (1) measure changes in self-reported music experiences after music rehabilitation or training, both at an individual and group level, and (2) identify areas of focus and rehabilitation needs for individual CI users. Specific instructions on how to use and score the questionnaire in each of those scenarios are provided in a separate instruction sheet in the Supplementary Material of this article.

## 3. Results

### 3.1. Study Characteristics

In total, 11 original research articles were included in the present review (Figure 1). Three of the studies were translations and validations of the MuRQoL to non-English languages [25,26,27] while eight studies reported applications of the original English version of the MuRQoL or its translations [28,29,30,31,32,33,34,35]. Studies were conducted in four different countries (US, Italy, Turkey, Spain) and published between 2021 and 2025. All studies reviewed used CI patients as their main population, although in Fowler (2021) the questionnaire was also completed by Hearing Aid (HA) users. Eight studies used adult participants, whereas, in the rest of the studies, age groups varied starting from as young as 11 years old [34]. The primary focus across studies was the assessment of musical perception and QoL, but the specific aim of each study varied depending on the setting and context. Most studies followed a cross-sectional design, with only one non-randomized controlled trial (NRCT) reported [28]. Interventions commonly included audiological evaluations, music perception tests, and surveys, often comparing CI patients to normal hearing (NH) controls or alternative CI groups. Concerning CI devices, Lassaletta et al. 2025 used the Synchrony ST Flex28 electrode array (MED-EL, Innsbruck, Austria) and two other studies reported using MED-EL devices (model and electrode details not specified) [27,30,33]. The remaining studies did not provide any information in this regard. Outcomes were evaluated using various tools such as audiological battery tests, Comprehensive Auditory Music Perception (CAMP), Nijmegen Cochlear Implant Questionnaire (NCIQ), Mistuning Perception Test (MPT), Music Quality Perception Platform (MuQPP), Duration Pattern Test (DPT), Frequency Pattern Test (FPT), Music Use and Music Utility (MUMU). The general characteristics of the reviewed studies, the purpose of MuRQoL use, study design, population, intervention, comparison and outcomes are summarized in Table 2.

### 3.2. Translation and Validation

Three studies were conducted with the objective to translate and validate the MuRQoL into Italian, Spanish and Turkish, as summarized in Table 3. All three studies used traditional psychometric techniques for validation, similarly to the original instrument. Exploratory Factor Analysis (EFA) resulted in two factors (perception, engagement), as in the original English version. Authors retained the same number of questions as the original version with the exception of Akbulut et al. (2022), who retained 17 Frequency items and 18 Importance items, as per the results of factor analysis. In all three translations, for both Part I and II, all scales and subscales had excellent internal consistency (Cronbach α > 0.80). Generally, authors used data collected by CI users for validation. However, Frosolini et al. (2022) only used responses from NH adults for their factor analysis and internal consistency [27]. The Turkish and Spanish versions had excellent test–retest reliability for all scales and subscales (ICC > 0.80). No test–retest reliability assessment was reported for the Italian version. Convergent validity was confirmed by moderate to strong correlations (r = 0.242–0.645) between the Spanish MuRQoL Frequency Total scores and Meludia platform performance [26]. All three validation studies used Confirmatory Factor Analysis (CFA) and the ‘known groups’ method to confirm construct validity. Akbulut et al. (2022) reported statistically significant differences between the CI and NH groups for the Frequency scale. Frosolini et al. (2022) showed significant differences between NH amateur musicians, NH sport practitioners and CI users [27]. The authors also found correlations of MuRQoL scores with musical studies, musicians and age. Frosolini et al. recently published a correction to the original article where they revised item 3 of the questionnaire to better convey the intended meaning [27]. Specifically, they revised the original wording asking about loudness (“differenze di dinamica” in Italian) into “differenze nel tono musicale”, referring to pitch differences. In the correction study, they also collected 52 responses to the questionnaire with the revised item and conducted EFA to validate the updated version that should now be used in all subsequent applications. Zuazua-Gonzalez et al. (2024) reported no significant differences between CI and NH adults for the Importance scale, confirming the assumption that, despite poorer perception, CI users consider music equally important as their NH peers. Moreover, Frosolini et al. (2022) reported correlations of MuRQoL scores with musical studies, musical profession and age, while Zuazua-Gonzalez et al. (2024) found that frequency scores showed high correlations with all scores on Meludia, with the highest correlations in Stable/unstable, Melody and Density. They reported notable discriminative capacity using the five Meludia Discovery module tasks which they treated as “gold standard” for this purpose.

### 3.3. Application

The eight studies that applied the MuRQoL highlight the diverse employment of the questionnaire across different populations, methodologies, and settings, as summarized in Table 4. Six studies used the MuRQoL to compare CI users with NH adults, CI users with other groups of adults with HL or different groups of CI users. Gökay et al. (2024) explored the effect of residual hearing using the Turkish version, showing improved temporal auditory skills and musical perception subskills in CI users with residual hearing (compared to those without), confirming its influence on QoL. Lassaletta et al. (2025) compared CI fitting techniques using the Spanish version, finding no significant differences overall but slightly higher Frequency-Perception scores in dynamic fitting (DF) compared to Anatomy Based Fitting (ABF) of Synchrony ST Flex28 CI (MEDEL, Innsbruk, Austria) users. DF refers to the standard method for CI programming, where frequency bands are distributed logarithmically across the cochlea without considering individual anatomical differences. ABF uses imaging (CAScination, AG, Bern, Switzerland) to align electrode frequency settings with the natural tonotopic organization of the cochlea, aiming to reduce frequency mismatches. ABF showed specific advantages in musical tasks, particularly in processing multiple simultaneous sounds, as demonstrated in the Density category of the Meludia platform. This points to DF’s potential advantages in processing complex auditory inputs, warranting further exploration. Akbulut et al. (2025) found that NH participants scored higher than CI users on Frequency [29]. Authors also reported associations between MuRQoL scores and demographics, age, music-related and audiological characteristics.

The remaining three studies also explored associations between the MuRQoL and objective music perception tests, in addition to testing group comparisons. Kosemihal et al. (2023) applied the Turkish version to explore mistuning perception, demonstrating significant correlations between Frequency scores and the MPT, underscoring the challenges CI users face in perceiving structural harmony in music. Calvino et al. (2023) compared Spanish-speaking NH and CI populations and showed that NH users scored higher in Frequency but similarly in Importance in the Spanish version of MuRQoL. Strong correlations with Meludia platform tasks confirmed its capability to capture nuanced music perceptions across groups [33]. Meludia is an interactive music education and training platform designed to evaluate, develop and enhance various musical skills. It employs gamified and intuitive exercises that focus on core aspects of music perception, including pitch, rhythm, harmony and melody. Meludia’s tasks are adaptable and suitable for users with varying levels of musical expertise, from beginners to advanced learners, and have been applied in combination with MuRQoL in three different studies to date [28,30,33]. In her PhD thesis, Fowler (2021) utilized the instrument as a complement when evaluating speech-in-noise and music perception in NH and CI groups to assess its utility as a proxy, as well as to test its sensitivity in distinguishing groups of different levels of HL. While MuRQoL was indeed sensitive to some expected group differences, i.e., NH participants and HA users outperformed CI users, findings indicated no significant relationships between self-reported music perception and objective music perception scores for CI users, which did not confirm the ability of MuRQoL to be used as a proxy measure of music perception. However, these findings suggest that the MuRQoL is appropriate and effective for younger as well as older adults and HA users in addition to CI recipients, thus proving the instrument’s wider applicability. A corresponding journal article from the same research group that reported part of the work of the PhD thesis mentioned the use of the MuRQoL in the study but did not actually report any details of how it was used [36].

In the unique longitudinal study reported to date, Frosolini (2024) employed the MuRQoL to test music training interventions, namely 1 month use of the Meludia app at home, and observed significant improvements in Frequency and Perception scales, with 57% of participants reporting meaningful changes. This finding supports auditory music training (AMT) as a promising tool for enhancing QoL in CI users but also the MuRQoL utility in pre- to post-training evaluation of music training. Notably, two studies used the matrix proposed in the original ‘Instructions for use’ combining ‘Frequency’ and ‘Importance’ scores to understand the impact of music on QoL and identify individual rehabilitation needs. Akbulut et al. (2025) plotted MuRQoL scores on the matrix for each question for both CI users and NH participants to determine the normal, high and low scores for frequency and importance. As in the original instructions, the Strong Negative Impact (SNI) area of the matrix indicated “critical” areas for clinical rehabilitative purposes. Frosolini et al. (2022) further introduced the Rehab Factor, an innovative metric that quantifies music rehabilitation needs as the 0–100 difference between the MuRQoL Frequency and Importance scores [34]. The authors also reported significant correlations with the Nijmegen Cochlear Implant Questionnaire (NCIQ) [37]. Taken together, these findings confirm MuRQoL’s potential to help identifying individual music rehabilitation needs.

### 3.4. Administration and Scoring

MuRQoL was administered both in person and online, confirming validity of the instrument in both formats (Table 2 and Table 3). Several authors reported converting the MuRQoL scores to the 0–100 scale as per the original instructions, while others used exclusively the 1–5 Likert scale. All studies utilized the complete MuRQoL questionnaire by calculating scores for both the Frequency and Importance scales, as well as the Perception and Engagement subscales. In addition, Frosolini et al. plotted the MuRQoL scores on the matrix, provided a decision tree and score calculator and further calculated the questionnaire-derived Rehab Factor, as explained above [26,34].

## 4. Discussion

In the last decade, the research landscape of music and CIs has changed significantly. The patient perspective is considered critical in driving research questions and design [11]. There is now a better understanding of the cues that are available to CI listeners and strategies to improve the perception and enjoyment of music for CI users. Additionally, novel music rehabilitation tools, such as music training programs, have been designed to enhance music enjoyment [13]. These interventions have shown potential to significantly improve multiple measures of music perception in experimental settings [38]; however, high-quality evidence for their effectiveness is still lacking, partly due to the absence of uniform outcome measures. As a result, music rehabilitation tools that could strongly benefit patients are not widely adopted by professionals. The MuRQoL questionnaire, introduced in 2017, serves as a valid and reliable self-report measure to evaluate music experience, importance and impact on QoL in CI users. This review aimed to explore the application of the instrument, assess to what extent its goals have been achieved and make recommendations for moving forward.

Performance variability among CI users in music perception and enjoyment is well documented, with certain groups consistently outperforming others due to various influencing factors. For example, CI users with residual hearing in the contralateral ear, such as bimodal users or single-sided deafness, tend to perform better in music perception tasks due to the acoustic cues provided by the non-implanted ear, which enhance pitch resolution and timbre perception [39]. These users also showed higher MuRQoL scores [35]. Acoustic advantages facilitate better recognition of melody and rhythm compared to those relying solely on electrical stimulation [40]. Pre-implantation musical experience also plays a significant role in determining music perception outcomes, with studies consistently showing that individuals with strong musical backgrounds prior to implantation outperform their peers in music perception tasks, likely due to their familiarity with musical structures and neural pathways already primed for music processing [41]. Accordingly, Frosolini et al. (2022) reported correlations between scores of the Italian version of the MuRQoL (MuRQoL-It) and individual musical background [26]. Furthermore, post-implantation music training has been found to enhance auditory processing and compensate for the inherent physical limitations of CI devices [17,28]. Age at implantation and the duration of auditory deprivation are additional critical factors. Younger CI users, particularly those implanted shortly after the onset of hearing loss, typically achieve better outcomes, benefiting from greater neural plasticity and shorter periods of auditory deprivation [42]. By contrast, long-term deafened individuals may face more challenges in adapting to the electrical stimulation provided by the CI and interpreting complex musical nuances, even though this does not necessarily correlate with musical appreciation as revealed by MuRQoL [5,28,43]. Variability is also affected by differences in CI technology and fitting strategies [44]. Notably, ABF has demonstrated potential in enhancing subjective music perception, as reflected in improved MuRQoL scores and performance on tasks like distinguishing simultaneous sounds and identifying harmonic structures [30]. These findings, supported by the robust body of literature, underscore the importance of tailored interventions and rehabilitative strategies guided and evaluated not only with objective tests, but also through the use of the MuRQoL questionnaire. The heterogeneity of clinical scenarios and scientific results also highlights the critical need for personalized approaches in addressing the diverse challenges of music perception and enjoyment in individuals with HL.

### 4.1. Lessons Learned

This review has demonstrated several novel aspects of MuRQoL use. The instrument is now available in four languages and has been validated in five different countries across diverse cultural and linguistic contexts. It has been implemented with a range of people with HL, specifically HA users, unilateral and bilateral CI users as well as younger CI users (>11 years old) [28,33,34]. The successful administration of the instrument—originally designed and validated for adult CI users—to these groups, supports its potential for wider use in the field and diverse future adaptations. Interestingly, Calvino et al. (2023) reported using the MuRQoL as a basis to develop a paediatric music questionnaire. This review underscores the growing adoption of MuRQoL and its relevance in both research and clinical practice. Furthermore, emerging trends include integrating the questionnaire with other assessment tools like the NCIQ, MUMU, CAMP and the Meludia platform. This way, MuRQoL’s self-reported personal and subjective experience can be complemented and combined with psychophysical measures less influenced by individual biases, memory recall issues and response tendencies (such as social desirability or over/underestimating abilities) which are inherent in any self-report tool. The MuRQoL has also been applied in various research and clinical settings, with a single longitudinal NRCT study suggesting that the tool can indeed be utilized to measure changes after music rehabilitation. However, while the instrument correlates with music perception tests (e.g., ‘mistuning’ tests), its ability to serve as a proxy was not fully supported [31,32]. Another novel finding from this review is the development of the Rehab Factor, which deserves further investigation to confirm its predictive power but is particularly important for identifying rehabilitation needs [28]. Normal, high and low thresholds for Frequency and Importance were also defined, facilitating the identification of “critical” rehabilitation areas using the matrix.

The review also confirmed and further supported MuRQoL’s use with various subgroups of adult CI users (e.g., pre/post-lingual, younger/older, etc.). It highlighted the instrument’s flexibility, as it can be applicable in online and paper versions, the utility of frequency and importance scores and the conversion of 1–5 scores to a 0–100 scale. Importance ratings, in particular, can be used to capture music preferences and different levels of education and training affecting the comparability of responses across individuals. In previous music questionnaires, this was typically assessed with optional musical background sections that were often difficult to quantify or score. Moreover, the use of the matrix to plot individual scores and identify critical areas for music rehabilitation has been further supported by the introduction of the ‘Rehab Factor’. Correlations of the instrument with health-related QoL measures (e.g., NCIQ) further validate the QoL component of MuRQoL and its potential to provide insights into the impact of music experiences on physical, emotional and social well-being [28,34]. This stresses MuRQoL’s potential to shed light on the physical, emotional and social impact of CI users’ music experiences, which has been mostly overlooked to date [15].

### 4.2. Access and Naming of Updated MuRQoL Versions

The original English version of the MuRQoL and the three translations are provided in the Supplementary Material of this article. This ensures researchers and clinicians have immediate access to the validated instruments, facilitating their use in both clinical and research settings. The authors aim to encourage further applications of MuRQoL, particularly in assessing music perception and QoL in CI users. The availability of this resource also highlights the importance of promoting standardized tools for global research collaborations and data comparability. In all MuRQoL versions, a minor change has been made in item 13 of Part I and II replacing DVD and computer with laptop, tablet or phone. This change was deemed necessary by the authors to reflect technologies available and used nowadays. Additional minor adaptations have been made in the original English version to adapt to American English spelling, as the instrument is now widely used in USA as well as the UK. Although the MuRQoL is freely available, we recommend as best practice that researchers seek permission from the corresponding author of each version before use or translation. A template can also be provided upon request to preserve a uniform formatting and layout across future translations.

For consistency, we advise that the original 2017 English version is referred to as MuRQoL or MuRQoL-En and all current and future validated translations are named as follows: MuRQoL-[initials of language], e.g., MuRQoL-It, MuRQoL-Tr, MuRQoL-Sp. This will facilitate references to the translations and will ensure no confusion is made with the original English version. Additional acronyms in the native languages of the translations can also be used, such as MUSQUAV in the case of Italy. Moreover, different acronyms have been used in the literature to refer to MuRQoL’s components and scores. For instance, the Total score, Perception and Engagement subscales have been referred to as FS, FS_MP_ and FS_ME_ by Akbulut et al. (2025) or as F-T, F-P and F-E by Frosolini et al. (2024) [28,29]. For consistency, we recommend that the acronyms in Table 1 are used by all authors.

### 4.3. Recommendations for Use

In 2017, a document with instructions was developed to accompany the English version of the MuRQoL that was in the public domain via the University of Southampton repository and was also provided directly to authors who expressed an interest. The document recommended two different ways to use the questionnaire based on the original publication (assessment of post-intervention group/individual changes or identification of individual rehabilitation needs), as well as converting 1–5 scores to a 0–100 scale. The document also included the Smallest Detectable Change (SDT) for each scale and subscale score that should be used when assessing music interventions. Most recommendations were subsequently followed by researchers to a lesser or greater extent, with the exception of assessing individual changes after music rehab as shown in the results section above. In addition, researchers used the MuRQoL in a number of other ways not included in the instructions (e.g., assessment of group differences, use with HA users and younger CI users, calculation of a rehab factor) which has provided further insights into the applicability and future potential of the tool, and has opened avenues for future work and implementation. Based on these novel findings, we make recommendations for the future use and reporting of the MuRQoL throughout this article. We emphasize standardizing translation and validation processes and optimal practical adoption, ensuring greater consistency in future applications. However, our recommendations should by no means restrict researchers from using or adapting the questionnaire in another way.

### 4.4. Anecdotal Evidence

In addition to published findings, this review provided insights into MuRQoL’s broader potential. We identified 12 non-English publications (in Portuguese, Italian, Korean, Turkish, Chinese, Thai and French), including a validated Korean version of MuRQoL published with an English abstract [45]. However, the language barrier limited a full review of these works. Future efforts should encourage authors to publish their work in English or international journals to facilitate knowledge sharing and enhance accessibility. Moreover, the authors are aware of unpublished or ongoing work related to MuRQoL, such as the development of a paediatric version, which reflects the tool’s growing global adoption. Several researchers have sought permission to translate and adapt the questionnaire into languages such as Hebrew, Chinese, German, Thai and Filipino. Many original articles are forthcoming and will be an important contribution in the near future. Although these documents fall outside this review’s scope as they did not meet the criteria for inclusion, they nonetheless demonstrate MuRQoL’s broad applicability across diverse linguistic and cultural settings. These works also reflect its utility and relevance in assessing music perception and QoL among CI users globally. The availability of such publications highlights the reach of MuRQoL beyond English-speaking populations and its potential as a universal tool, reinforcing its value for future adaptations and validations in various international contexts.

**Figure 2:**
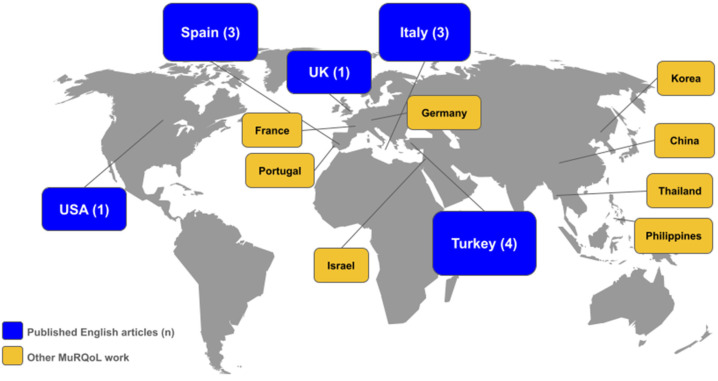
MuRQoL adoption worldwide. In blue: locations of MuRQoL application in the 11 articles included in the present review in addition to the original validation study in the UK [19]. In brackets the number of published articles per country. In yellow: potential geographical spread of MuRQoL considering ongoing work, expressions of interest, non-English articles found or permissions for translation granted. Translations may also cover other counties where the language is spoken beyond the country of validation.

The MuRQoL shows great promise as a standard tool for assessing music perception and its impact on the QoL in CI users. Its multidimensional approach, which evaluates subjective music appreciation alongside psychophysical tasks, provides a comprehensive framework for understanding the role of music in CI users’ lives, thus addressing an important knowledge gap in the field of HL and music. By offering quantifiable and reproducible metrics, the MuRQoL bridges the gap between subjective experiences and clinical outcomes, paving the way for its integration into routine assessments. Moreover, its adaptability to various languages and cultural contexts further enhances its potential as a globally recognized standard (Figure 2).

### 4.5. Limitations and Gaps

Among the methodological limitations of this review is the use of two databases, which may have restricted the breadth of studies identified. This choice was influenced by the scope and available resources but could have resulted in missing relevant publications, particularly those in less commonly indexed journals. The use of a simple search strategy may have further limited the comprehensiveness, as more complex queries might have captured a wider array of studies. Additionally, the restriction to translation or implementation studies excluded broader applications that might provide valuable insights into the potential utility of MuRQoL. Also, the exclusion of non-English publications and unpublished work likely resulted in missed insights, particularly from regions where the MuRQoL is being adopted, such as parts of Asia, South America, and the Middle East. These areas may have valuable data on how the tool is being adapted and used in diverse cultural and linguistic settings, which could inform its broader applicability and future development. However, these were partly imposed by the scope of this review and the relatively short lifetime of the MuRQoL as a research and clinical instrument. We are confident that more ongoing work will be published in the future to enable a more complete review. Beyond methodological limitations and despite the MuRQoL developments and novel findings from the articles of this review, a number of issues and knowledge gaps remain to be addressed. First, the included studies predominantly employ cross-sectional designs, limiting insights into the longitudinal applicability of MuRQoL for tracking changes over time or evaluating interventions. Future studies could address this gap by designing longitudinal research that follows CI users over extended periods to observe how their music-related QoL evolves. Additionally, intervention-based research, such as assessing the impact of music training or rehabilitation programs on MuRQoL scores, could provide valuable data on the tool’s responsiveness to therapeutic interventions. Second, variability in administration methods (paper vs. online) and scoring approaches across studies introduce heterogeneity, which may impact comparability of results. Additionally, some studies did not report critical psychometric properties (such as test–retest reliability), used only NH data for validation or omitted details about scoring and data analysis methods. Finally, the absence of a standardized naming convention for translations and the limited use of MuRQoL in routine clinical practice highlight challenges in accessibility and broader adoption. Without consistent naming conventions and streamlined protocols, researchers and clinicians may have faced difficulties in accurately interpreting or applying findings, which could hinder the integration of MuRQoL into broader clinical and research frameworks. Addressing these limitations in future research is crucial for optimizing the instrument’s utility and ensuring consistency across studies.

### 4.6. Future Directions

Future research could focus on developing child-friendly versions of the MuRQoL in all existing languages to address the specific needs of younger CI users. Features such as simplified language tailored to a child’s reading level, engaging visuals like pictograms or cartoons and interactive response formats (e.g., smiley faces for Likert scales) could make the tool more accessible. Additionally, including questions that reflect children’s everyday musical experiences, such as school music activities or listening to songs during play, would enhance its relevance. Pilot testing in diverse paediatric populations would ensure the tool’s reliability and effectiveness in capturing music-related QoL in younger CI users. Although Calvino et al. (2023) used MuRQoL as a basis for the development of the MuQPP, a dedicated paediatric version of MuRQoL in any language is still ongoing work. Validating this tool with prelingual and post-lingual children, as well as typically hearing peers, could provide valuable insights into how music impacts their QoL and guide tailored rehabilitation programs. The use of the current adult versions of the MuRQoL with CI users as young as 11 years old presented in this review can provide valuable insights into the adaptation of the instrument for children. Additionally, creating a shortened MuRQoL version would enhance its practicality in clinical settings by focusing on core items to reduce completion time while maintaining reliability. Alternatively, the questionnaire could be administered using a novel adaptive procedure whereby the importance question is asked as a filter question first for each item and only items scored rated as important are then assessed for frequency. This way, the tool could serve as a quick screening measure to identify individuals who might benefit from more detailed assessment or targeted music rehabilitation. Expanding MuRQoL’s application to HA users would also extend its relevance to a broader population. Validating the tool for this group could offer new perspectives on their music-related experiences and challenges, supporting personalized interventions to enhance their engagement with music. Finally, further data collection should establish thresholds to categorize a CI user’s music experience as “good”, “moderate”, or “poor”. This will enhance MuRQoL’s interpretability and improve its clinical and research utility.

## 5. Conclusions

The MuRQoL has been validated and used internationally in the UK, US, Italy, Turkey and Spain, which shows its growing global relevance. The review highlights its strong psychometric properties across languages and versatility in both clinical and research settings. We have identified several gaps and opportunities for future work, such as the limited longitudinal studies which restrict the comparability of results and the need for a paediatric version and validation for other populations with HL, such as HA users, to expand its utility. Efforts to standardize translations, scoring, and naming conventions are critical for fostering consistency and access. The MuRQoL positions itself as a standard for assessing music experiences of CI users and impact on QoL. Its integration into routine clinical assessments and rehabilitation programs holds the potential to significantly improve holistic care for individuals with HL, paving the way for a deeper understanding of music’s integral role in enhancing emotional and social well-being.

## Figures and Tables

**Table 1:** The MuRQoL structure and derived scores.

The Music-Related Quality of Life Measure (MuRQoL)	
Part I. ‘Frequency’ (18 items)	Part II. ‘Importance’ (18 items)
‘Frequency Perception’ (FP) score, 11 items ‘Frequency Engagement’ (FE) score, 7 items ‘Frequency Total’ (FT) score	‘Importance Perception’ (IP) score, 11 items ‘Importance Engagement’ (IE) score, 7 items ‘Importance Total’ (IT) score

**Table 2:** General characteristics and PICOS of studies included in the present review.

First Authors (Year) [Ref.]	Country	Rationale for Inclusion	MuRQoL Purpose of Use	Population	Intervention	Comparison	Outcome	Study Design
Fowler (2021) [31]	USA	Application	Understand differences/assess sensitivity; Assess utility as proxy	CI	Audiological evaluation, music perception test and survey	NH and other groups (e.g., pre-/post-lingually deaf, uni-/bi-lateral, HA users)	Audiometry battery; CAMP; MurQoL	Cross-sectional
Akbulut (2022) [25]	Turkey	Translation and cross-cultural validation		CI	Survey or N/A	NH	Goodness-of-fit indices; MuRQoL	Cross-sectional
Frosolini (2022) [27]	Italy	Translation and cross-cultural validation		CI	Audiological evaluation, survey or N/A	NH	Audiometry battery; Goodness-of-fit indices	Cross-sectional
Frosolini (2022) [34]	Italy	Application	Test utility to guide music rehab	CI	Audiological evaluation, survey	NCIQ	Audiometry battery; MurQoL; NCIQ	Cross-sectional
Kosemihal (2023) [32]	Turkey	Application	Investigate the mistuning perception and compare with music-related QoL.	CI	Music perception test and survey	NH	MPT; MuRQoL	Cross-sectional
Calvino (2023) [33]	Spain	Application	Evaluate different music tasks through the Meludia platform	CI	Music perception test and survey	NH; three CI-age cohorts	Meludia; MuQPP; MurQoL;	Cross-sectional
Akbulut (2025) [29]	Turkey	Application	Assess impact of music on QoL	CI	Survey	NH	MuRQoL	Cross-sectional
Gökay (2024) [35]	Turkey	Application	Assess effect of residual hearing	CI with residual hearing	Auditory processing test and survey	CI without residual hearing	FPT; DPT; MuRQoL	Cross-sectional
Zuazua-Gonzalez (2024) [26]	Spain	Translation and cross-cultural validation		CI	Survey or N/A	NH	Goodness-of-fit indices	Cross-sectional
Frosolini (2024) [28]	Italy	Application	Evaluate Meludia music rehab	CI	Music rehabilitation training	CI (no training)	Audiometry battery; MurQoL; NCIQ	NRCT
Lassaletta (2025) [30]	Spain	Application	Assess effects of different fitting techniques	CI with designed fitting technique	Music perception test and survey	CI (routine care)	Meludia; MuRQoL; MUMU	Cross-sectional

Abbreviations: CI: Cochlear Implant; NH: Normal Hearing; MuRQoL: Music-Related Quality of Life; CAMP: Comprehensive Auditory Music Perception; NCIQ: Nijmegen Cochlear Implant Questionnaire; MPT: Mistuning Perception Test; MuQPP: Music Quality Perception Platform; DPT: Duration Pattern Test; FPT: Frequency Pattern Test; NRCT: Non-Randomized Controlled Trial; MUMU: Music Use and Music Utility.

**Table 3:** Results extracted from the MuRQoL cross-cultural translations and validations.

First Author (Year) [Ref.]	Target Language	Population	Administration	Factor Analysis	Test–Retest Reliability	Internal Consistency	Validity
Frosolini (2022) [27]	Italian	180 NH adults (Mage: 34) 35 CI users (Mage: 60)	35 CI users on paper 180 NH online	With NH participants only. EFA/CFA: 2 factors, perception, engagement	Not reported	With NH participants only Excellent (Cronbach α > 0.80) for FREQ and IMP	Significant differences between: NH Amateur Musicians, NH Sport practitioner and CI users. Correlations with musical studies, musicians and age.
Akbulut (2022) [25]	Turkish	161 CI users (Mage: 30) 162 NH adults (Mage: 30)	221 online 62 in person	2 factors: perception, engagement 17 Frequency items 18 Importance items	Excellent at 2 weeks (ICC > 0.80)	Excellent (Cronbach α > 0.80) for all scales and subscales	Significant differences between CI and NH group for Frequency scale.
Zuazua-Gonzalez (2024) [26]	Spanish	55 CI users and 74 NH peers ≥ 17 years	In-person and online	EFA: 2 factors explained both scales with high correlation between them.	95/129 CI users (74%) at 15 days. Very high ICC (>0.8) for all scales and subscales.	Very high Cronbach α (>0.8) for all scales and subscales.	Significant differences in Frequency scores between NH and CI users and high correlations with Meludia tasks, highlighting strong discriminative capacity and convergent validity.

Abbreviations: NH; Normal Hearing; CI; Cochlear Implant; Mage; Mean Age; EFA; Exploratory Factor Analysis; CFA; Confirmatory Factor Analysis; FREQ; Frequency; IMP; Importance; ICC; Intraclass Correlation Coefficient; sMuRQoL; Spanish Music-Related Quality of Life Questionnaire.

**Table 4:** Results extracted from the retrieved original research publications reporting MuRQoL applications.

Study (Year) [Ref.]	MuRQoL Methodology/Population	Results	Conclusion
Fowler (2021) [30]	In person survey; 10 NH, 60 adult CI users; scores converted to 0–100	Young NH group outperformed post-lingual CI group and young post-lingual HA users outperformed CI users in MuRQoL. MuRQoL scores on the ability to identify musical instruments significantly correlated with the respective objective scores.	MuRQoL was sensitive to some group differences with the expected large effect sizes. Limited ability of MuRQoL to be used as a proxy for actual objective measures.
Frosolini (2022) [33]	Italian version in person and online survey; 73 CI > 11 years	Correlation between MUSQUAV (MuRQoL-It) and NCIQ; developed Rehab Factor.	MUSQUAV (MuRQoL-it) can improve CI audiological evaluations; Rehab Factor can guide interventions.
Kosemihal (2023) [32]	Turkish version in person survey; 16 CI, 16 NH	Significant correlation of MuRQoL–FS with MPT; no correlation with MuRQoL–IS.	CI recipients experience limitations in perceiving harmony. Mistuning assessment should be considered in music-based auditory tests and interventions in CI recipients.
Calvino (2023) [33]	Spanish version in person survey; 39 CI, 39 NH	NH scored higher in FREQUENCY; IMPORTANCE scores similar; significant Meludia correlations.	MuRQoL captures nuanced perception across CI and NH populations.
Gökay (2024) [35]	Turkish version, in person survey; 40 CI (20–45 y); 2 groups according to absent (20 CI) or available (20 CI) residual hearing before implantation	Frequency and duration pattern recognition skills were significantly better in CI users with residual hearing. Significant correlations in terms of temporal and musical perception skills.	The presence of residual hearing before CI may affect temporal auditory processing skills. The integration of CI may affect temporal processing skills in adults and QoL.
Akbulut (2025) [29]	Turkish version online survey; 214 CI, 193 NH	NH scored higher on FS; music negatively impacted QoL in 31%, positively in 58% of CI users.	Identifying CI challenges improves music-related therapeutic effectiveness.
Frosolini (2024) [28]	Italian version in person and online survey; 21 CI (training), 19 CI (control)	Training improved Frequency Total and Perception; 57% meaningful changes in users post-training.	AMT shows potential for improving QoL in CI users.
Lassaletta (2025) [30]	Spanish version in person survey; 20 CI (10 DF, 10 ABF)	No significant overall differences; Frequency-Perception scores slightly higher in DF users.	ABF offers advantages in processing multiple sounds simultaneously. Further studies should explore additional benefits of ABF in musical skills in CI users.

Abbreviations: NH: Normal Hearing, CI: Cochlear Implant, NCIQ: Nijmegen Cochlear Implant Questionnaire, FS: Frequency Scale, IS: Importance Scale, MPT: Mistuning Perception Test, DF: Dynamic Fitting, ABF: Anatomy-Based Fitting, MuRQoL: Music-Related Quality of Life.

## Data Availability

All versions of the MuRQoL questionnaire reviewed are provided as Supplementary Material.

## Supplementary Materials

The following supporting information can be downloaded at: https://www.mdpi.com/article/10.3390/audiolres15020026/s1, S1: Original MuRQoL English version (MuRQoL-En); S2: MuRQoL Italian version (MuRQoL-It); S3: MuRQoL Turkish version (MuRQoL-Tr), S4: MuRQoL Spanish version (MuRQoL-Sp), S5: PRISMA Checklist.

## Abbreviations

The following abbreviations are used in this manuscript:

MuRQoL	Music-Related Quality of Life measure
QoL	Quality of Life
CI	Cochlear Implant
HL	Hearing Loss
PROM	Patient-Reported Outcome Measure
PICO	Participant Intervention Comparison Outcome
MuMu	Munich Music Questionnaire
IMBQ	Iowa Musical Background Questionnaire
UCMLQ	University of Canterbury Music Listening Questionnaire
FT	Frequency Total score
FP	Frequency Perception score
FE	Frequency Engagement score
IT	Importance Total score
IP	Importance Perception score
IE	Important Engagement score
NH	Normal Hearing
PRISMA	Preferred Reporting Items for Systematic Reviews and Meta-Analyses
HA	Hearing Aid
NRCT	Non-Randomised Controlled Trial
CAMP	Comprehensive Auditory Music Perception
NCIQ	Nijmegen Cochlear Implant Questionnaire
MPT	Mistuning Perception Test
MuQPP	Music Quality Perception Platform
DPT	Duration Pattern Test
FPT	Frequency Pattern Test
MUMU	Music Use and Music Utility
EFA	Exploratory Factor Analysis
ICC	Intraclass Correlation Coefficient
CFA	Confirmatory Factor Analysis
ABF	Anatomy-Based Fitting
DF	Dynamic Fitting
AMT	Auditory Music Training
SNI	Strong Negative Impact
SDT	Smallest Detectable Change
