# Supplementary material for: The Music-Related Quality of Life Measure (MuRQoL): A Scoping Review of Its Validation and Application
Source: Audiol Res. 2025 Mar 7;15(2):26. doi: 10.3390/audiolres15020026 (PMC11932307; doi:10.3390/audiolres15020026)
Supplement: Supplementary file 1 [file audiolres-15-00026-s001.zip › S1. MuRQoL-En v3.pdf]

The 'Music-Related Quality of Life' measure (MuRQoL-En)

Part I

This part of the questionnaire asks you about your music listening abilities, attitudes towards music and musical activities. Please answer the following questions by using one of the options: **1: Never, 2: Rarely, 3: Occasionally, 4: Frequently, 5: Always, N/A: Not Applicable**.

	MUSIC PERCEPTION	1	2	3	4	5	N/A
1.	Can you distinguish different rhythms in music?						
2.	Can you follow the melody in music (i.e. follow the melody of a song or a familiar tune)?						
3.	Can you hear differences in musical tone (i.e. how high or low music is)?						
4.	Can you recognize the words in songs?						
5.	Can you recognize the sounds of different musical instruments?						
6.	Can you hear the meaning of music (i.e. the emotion, why it was created or what message it is trying to get across)?						
7.	Can you hear music without effort or having to concentrate?						
8.	Can you recognize familiar music (e.g. a song, singer or tune)?						
9.	Can you judge the quality of a musical performance (e.g. singing or musical instrument playing)?						
10.	Do you feel confident that you hear music like other people do?						
11.	Does music sound in tune?						

	MUSIC ENGAGEMENT	1	2	3	4	5	N/A
12.	Do you enjoy music in noisy environments when no visual cues are available (e.g. at a party, at a restaurant or in the car over the engine/ road noise)?						
13.	Do you enjoy music on TV, laptop, tablet or on the phone?						
14.	Do you choose to have music on in the background while doing something else (e.g. while reading, painting, doing gardening, exercising or just relaxing)?						
15.	Do you listen to music while travelling (e.g. in the car)?						
16.	Do you choose to listen to new music (i.e. music that you have not heard before)?						
17.	Do you attend public music events (e.g. musicals, concerts or music festivals)?						
18.	Do you sing, play a musical instrument or whistle?						

© 2025 Giorgos Dritsakis

Part II

This part of the questionnaire asks you how importance the above music listening abilities, attitudes towards music and musical activities are for you. Please answer the following questions by using one of the options: **<u>1: Not important at all</u>**, **<u>2: Not very important</u>**, **<u>3: Somewhat important</u>**, **<u>4: Very important</u>**, **<u>5: Extremely important</u>**, **<u>N/A: Not Applicable</u>**.

MUSIC PERCEPTION	1	2	3	4	5	N/A
1. How important is it for you to be able to distinguish different rhythms in music?						
2. How important is it for you to be able to follow the melody in music (i.e. follow the melody of a song or a familiar tune)?						
3. How important is it for you to be able to hear differences in musical tone (i.e. how high or low music is)?						
4. How important is it for you to be able to recognize the words in songs?						
5. How important is it for you to be able to recognize the sounds of different musical instruments?						
6. How important is it for you to be able to hear the meaning of music (i.e. the emotion, why it was created or what message it is trying to get across)?						
7. How important is it for you to be able to hear music without effort or without having to concentrate?						
8. How important is it for you to be able to recognize familiar music (e.g. a song, singer or tune)?						
9. How important is it for you to be able to judge the quality of a musical performance (e.g. singing or musical instrument playing)?						
10. How important is it for you to feel confident that you hear music like other people do?						
11. How important is it for you to hear music that sounds in tune?						

MUSIC ENGAGEMENT	1	2	3	4	5	N/A
12. How important is it for you to enjoy music in noisy environments when no visual cues are available (e.g. at a party, at a restaurant or in the car over the engine/road noise)?						
13. How important is it for you to enjoy music on TV, laptop, tablet or on the phone?						
14. How important is it for you to have music on in the background while doing something else (e.g. while reading, painting, doing gardening, exercising or just relaxing)?						
15. How important is it for you to listen to music while travelling (e.g. in the car)?						
16. How important is it for you to listen to new music (i.e. music that you have not heard before)?						
17. How important is it for you to attend public music events (e.g. musicals, concerts or music festivals)?						
18. How important is it for you to sing, play a musical instrument or whistle?						

© 2025 Giorgos Dritsakis
