# Supplementary material for: The Music-Related Quality of Life Measure (MuRQoL): A Scoping Review of Its Validation and Application
Source: Audiol Res. 2025 Mar 7;15(2):26. doi: 10.3390/audiolres15020026 (PMC11932307; doi:10.3390/audiolres15020026)
Supplement: Supplementary file 1 [file audiolres-15-00026-s001.zip › S2. MuRQoL-It v3.pdf]

Questionario Musica e qualità della vita (MuRQoL-It)

Parte I

La prima sessione del questionario (denominata FREQUENZA) riguarda le tue capacità di percezione della musica e il tuo coinvolgimento in attività di ascolto e pratica musicale. Per favore rispondi a tutte le domande barrando il riquadro corrispondente ad una delle 6 possibili risposte: **1) mai; 2) raramente; 3) di tanto in tanto; 4) spesso; 5) sempre; NS) non saprei.**

PERCEZIONE MUSICALE	1	2	3	4	5	NS
1. Riesci a distinguere diversi ritmi musicali?						
2. Riesci a seguire una melodia (ad esempio la melodia di una canzone o di un motivo familiare)?						
3. Riesci a sentire le differenze nel tono musicale (cioè quanto è acuta o grave la musica)?						
4. Riesci a riconoscere le parole nelle canzoni?						
5. Riesci a distinguere il suono dei diversi strumenti musicali (violino, pianoforte, sassofono, chitarra...)?						
6. Riesci a percepire il significato della musica (cioè l'emozione, perché è stata creata, quale messaggio vuole comunicare)?						
7. Riesci a sentire la musica senza bisogno di sforzarti, senza doverti concentrare?						
8. Riesci a riconoscere una musica che ti è familiare (ad esempio una canzone, un cantante o una melodia)?						
9. Sai giudicare la qualità di una performance musicale (ad esempio il cantato o la parte strumentale)?						
10. Pensi di udire la musica come tutti gli altri?						
11. Percepisci come intonata la musica che ascolti?						

COINVOLGIMENTO MUSICALE	1	2	3	4	5	NS
12. Ti piace la musica in ambienti rumorosi (ad esempio ad una festa, al ristorante o in auto) in assenza di stimoli visivi?						
13. Ti piace ascoltare la musica in TV, computer, tablet o cellulare quando è possibile seguire la performance anche visivamente?						
14. Metti la musica in sottofondo mentre fai qualcos'altro (ad es. durante la lettura, la pittura, il giardinaggio, i lavori domestici, l'esercizio o semplicemente il relax)?						
15. Ascolti musica mentre viaggi (ad esempio in auto)?						
16. Ascolti musica nuova, che non hai mai sentito prima?						
17. Partecipi a eventi musicali (ad esempio musical, concerti o festival musicali)?						
18. Canti, suoni uno strumento musicale o fischietti quando sei da solo?						

Parte II

La seconda sessione del questionario (denominata IMPORTANZA) è composta dalle stesse domande della prima parte, le risposte in questo caso riguardano quanto sono importanti per te le capacità di percezione della musica e il coinvolgimento in attività di ascolto e pratica musicale. Per favore, rispondi a tutte le domande barrando il riquadro corrispondente ad una delle 6 possibili risposte: **1) irrilevante; 2) non molto importante; 3) rilevante; 4) molto importante; 5) estremamente importante; NS) non saprei.**

PERCEZIONE MUSICALE	1	2	3	4	5	NS
1. Quanto è importante per te riuscire a distinguere diversi ritmi musicali?						
2. Quanto è importante per te riuscire a seguire una melodia (ad esempio la melodia di una canzone o di un motivo familiare)?						
3. Quanto è importante per te riuscire a sentire le differenze nel tono musicale (cioè quanto è acuta o grave la musica)?						
4. Quanto è importante per te riuscire a riconoscere le parole nelle canzoni?						
5. Quanto è importante per te distinguere il suono dei diversi strumenti musicali (violino, pianoforte, sassofono, chitarra...)?						
6. Quanto è importante per te riuscire a percepire il significato della musica (cioè l'emozione, perché è stata creata, quale messaggio vuole comunicare)?						
7. Quanto è importante per te riuscire a sentire la musica senza bisogno di sforzarti, senza doverti concentrare?						
8. Quanto è importante per te riuscire a riconoscere una musica che ti è familiare (ad esempio una canzone, un cantante o una melodia)?						
9. Quanto è importante per te riuscire a giudicare la qualità di una performance musicale (ad esempio il cantato o la parte strumentale)?						
10. Quanto è importante per te la consapevolezza di udire la musica come tutti gli altri?						
11. Quanto è importante per te percepire come intonata la musica che ascolti (armonica, melodiosa)?						

COINVOLGIMENTO MUSICALE	1	2	3	4	5	NS
12. Quanto è importante per te apprezzare la musica in ambienti rumorosi (ad esempio ad una festa, al ristorante o in macchina) in assenza di stimoli visivi?						
13. Quanto è importante per te ascoltare musica su TV, computer, tablet o cellulare quando è possibile seguire la performance anche visivamente?						
14. Quanto è importante per te avere musica in sottofondo mentre fai qualcos'altro (ad esempio durante la lettura, la pittura, il giardinaggio, i lavori domestici, l'esercizio o semplicemente il relax)?						
15. Quanto è importante per te ascoltare musica mentre viaggi (ad esempio in auto)?						
16. Quanto è importante per te ascoltare musica nuova, che non hai mai sentito prima?						
17. Quanto è importante per te partecipare a eventi musicali (ad esempio musical, concerti o festival musicali)?						
18. Quanto è importante per te cantare, suonare uno strumento musicale o fischiettare quando sei da solo?						
