# Supplementary material for: The Music-Related Quality of Life Measure (MuRQoL): A Scoping Review of Its Validation and Application
Source: Audiol Res. 2025 Mar 7;15(2):26. doi: 10.3390/audiolres15020026 (PMC11932307; doi:10.3390/audiolres15020026)
Supplement: Supplementary file 1 [file audiolres-15-00026-s001.zip › S3. MuRQoL-Tr v2.pdf]

‘Müzikle İlgili Yaşam Kalitesi’ Anketi (MuRQoL-Tr)

Bölüm I

Anketin bu bölümünde müzik dinleme becerileriniz, müziğe ve müzik etkinliklerine yönelik tutumlarınız sorgulanmaktadır. Lütfen aşağıdaki sorulara şu seçeneklerden birini kullanarak cevap veriniz: **1: Hiç**, **2: Nadiren**, **3: Ara Sıra**, **4: Sık Sık**, **5: Her Zaman**, **U/D: Uygun Değil**.

MÜZİK ALGISI	1	2	3	4	5	U/D
1. Müzikteki farklı ritimleri ayırt edebiliyor musunuz?						
2. Müzikteki melodiyi takip edebiliyor musunuz (Ör: Bir şarkının ya da tanıdığınız bir ezginin melodisini takip etmek)?						
3. Müziğin tonundaki değişiklikleri duyabiliyor musunuz (Ör: Müziğin ne kadar alçak ya da yüksek perdeden olduğunu)?						
4. Şarkılardaki sözleri anlayabiliyor musunuz?						
5. Farklı müzik enstrümanlarının seslerini tanıyabiliyor musunuz?						
6. Müziğin anlamını duyabiliyor musunuz (Ör: Duyguyu, neden yazıldığını ya da hangi mesajı vermeye çalıştığını)?						
7. Çaba göstermeden ya da konsantre olmak zorunda kalmadan müziği duyabiliyor musunuz?						
8. Aşina olduğunuz müzikleri tanıyabiliyor musunuz (Ör: Bir şarkıyı, bir şarkıcıyı ya da bir ezgiyi)?						
9. Bir müzikal performansın kalitesini değerlendirebilir misiniz (Ör: Şarkı söylenmesi ya da bir müzik enstrümanının çalınması)?						
10. Müziği diğer insanlar gibi duyduğunuzdan emin misiniz?						
11. Müziği ahenkli duyuyor musunuz?						

MÜZİK ETKİNLİKLERİ	1	2	3	4	5	U/D
12. Görsel ipuçları olmadığında gürültülü ortamlarda müzik dinlemekten keyif alıyor musunuz (Ör: Bir partide, bir restoranda ya da motorun çalıştığı/yol gürültüsünün olduğu bir arabada)?						
13. TV, bilgisayar, tablet veya telefon ile müzik dinlemekten keyif alıyor musunuz?						
14. Başka bir şey yaparken arka planda müzik olmasını tercih ediyor musunuz (Ör: Okurken, resim yaparken, bahçeyle uğraşırken, egzersiz yaparken ya da sadece dinlenirken)?						
15. Seyahat ederken müzik dinliyor musunuz (Ör: Arabadayken)?						
16. Yeni müzikleri dinlemeyi tercih ediyor musunuz (Ör: Daha önce duymadığınız bir müziği)?						
17. Toplumsal müzik etkinliklerine katılıyor musunuz (Ör: Müzikallere, konserlere ya da müzik festivallerine)?						
18. Şarkı söylüyor musunuz, bir müzik enstrümanı ya da ıslık çalıyor musunuz?						

© 2025 Giorgos Dritsakis (Turkish Translation, Marmara University)

Bölüm II

Anketin bu bölümünde, yukarıda belirtilen müzik dinleme becerilerinin, müzik ve müzik etkinliklerine yönelik tutumların sizin için ne kadar önemli olduğu sorgulanmaktadır. Lütfen aşağıdaki sorulara şu seçeneklerden birini kullanarak cevap veriniz: **1: Hiç Önemli Değil**, **2: Çok Önemli Değil**, **3: Biraz Önemli**, **4: Çok Önemli**, **5: Fazlasıyla Önemli**, **U/D: Uygun Değil**.

MÜZİK ALGISI	1	2	3	4	5	U/D
1. Müzikteki farklı ritimleri ayırt edebilmek sizin için ne kadar önemlidir?						
2. Müzikteki melodiyi takip edebilmek sizin için ne kadar önemlidir (Ör: Bir şarkının ya da tanıdığınız bir ezginin melodisini takip etmek)?						
3. Müziğin tonundaki değişiklikleri duyabilmek sizin için ne kadar önemlidir (Ör: Müziğin ne kadar alçak ya da yüksek perdeden olduğunu)?						
4. Şarkılardaki sözleri anlayabilmek sizin için ne kadar önemlidir?						
5. Farklı müzik enstrümanlarının seslerini tanıyabilmek sizin için ne kadar önemlidir?						
6. Müziğin anlamını duyabilmek sizin için ne kadar önemlidir (Ör: Duyguyu, neden yazıldığını ya da hangi mesajı vermeye çalıştığını)?						
7. Çaba göstermeden ya da konsantre olmak zorunda kalmadan müziği duyabilmek sizin için ne kadar önemlidir?						
8. Aşina olduğunuz müzikleri tanıyabilmek sizin için ne kadar önemlidir (Ör: Bir şarkıyı, bir şarkıcıyı ya da bir ezgiyi)?						
9. Bir müzikal performansın kalitesini değerlendirebilmek sizin için ne kadar önemlidir (Ör: Şarkı söylenmesi ya da bir müzik enstrümanının çalınması)?						
10. Müziği diğer insanlar gibi duymak sizin için ne kadar önemlidir?						
11. Ahenkli bir müziği duymak sizin için ne kadar önemlidir?						

MÜZİK ETKİNLİKLERİ	1	2	3	4	5	U/D
12. Görsel ipuçları olmadığında gürültülü ortamlarda müzik dinlemekten keyif alabilmek sizin için ne kadar önemlidir (Ör: Bir partide, bir restoranda ya da motorun çalıştığı/yol gürültüsünün olduğu bir arabada)?						
13. TV, bilgisayar, tablet veya telefon ile müzik dinlemekten keyif alabilmek sizin için ne kadar önemlidir?						
14. Başka bir şey yaparken arka planda müzik olması sizin için ne kadar önemlidir (Ör: Okurken, resim yaparken, bahçeyle uğraşırken, egzersiz yaparken ya da sadece dinlenirken)?						
15. Seyahat ederken müzik dinlemek sizin için ne kadar önemlidir (Ör: Arabadayken)?						
16. Yeni müzikleri dinlemek sizin için ne kadar önemlidir (Ör: Daha önce duymadığınız bir müziği)?						
17. Toplumsal müzik etkinliklerine katılmak sizin için ne kadar önemlidir (Ör: Müzikallere, konserlere ya da müzik festivallerine)?						
18. Şarkı söylemek, bir müzik enstrümanı ya da ıslık çalmak sizin için ne kadar önemlidir?						

© 2025 Giorgos Dritsakis (Turkish Translation, Marmara University)
