# Supplementary material for: The Music-Related Quality of Life Measure (MuRQoL): A Scoping Review of Its Validation and Application
Source: Audiol Res. 2025 Mar 7;15(2):26. doi: 10.3390/audiolres15020026 (PMC11932307; doi:10.3390/audiolres15020026)
Supplement: Supplementary file 1 [file audiolres-15-00026-s001.zip › S4. MuRQoL-Sp v2.pdf]

El Cuestionario de Calidad de Vida Relacionado con la Música (MuRQoL-Sp)

Parte I

La primera parte del cuestionario incluye una serie de preguntas sobre su capacidad de escuchar música, su actitud hacia la música y sus actividades musicales. Por favor, conteste a las preguntas marcando una de las siguientes opciones: **1: nunca**, **2: pocas veces**, **3: a veces,** **4: con frecuencia**, **5: siempre**, **N/A: no aplicable**.

PERCEPCIÓN MUSICAL	1	2	3	4	5	N/A
1. ¿Puede distinguir distintos ritmos musicales?						
2. ¿Puede seguir una melodía musical (es decir, seguir la melodía de una canción o de una pieza musical familiar)?						
3. ¿Puede diferenciar tonos musicales (es decir, tonos agudos y graves)?						
4. ¿Puede reconocer la letra de las canciones?						
5. ¿Puede reconocer los sonidos de diferentes instrumentos musicales?						
6. ¿Puede escuchar el significado de una pieza musical (es decir, la emoción, la razón por la que fue compuesta o el mensaje que intenta transmitir?)						
7. ¿Puede escuchar música sin esfuerzo y sin la necesidad de concentrarse?						
8. ¿Puede reconocer una melodía familiar (p.ej. una canción, un/a cantante, una pieza musical)?						
9. ¿Puede reconocer una melodía familiar (p.ej. una canción, un/a cantante, una pieza musical?						
10. ¿Tiene la sensación de que escucha música igual que otras personas?						
11. ¿Le suena la música afinada?						

DEDICACIÓN A LA MÚSICA	1	2	3	4	5	N/A
12. ¿Disfruta de la música en entornos ruidosos si no hay referencias visuales (p.ej. en una fiesta, en un restaurante o en el coche con el ruido del motor o de la calle de fondo)?						
13. ¿Disfruta de la música en la televisión, en el portátil, en la tablet o en el teléfono?						
14. ¿Pone usted música de fondo durante la práctica de actividades como la lectura, la pintura, la jardinería, el ejercicio físico, etc. o mientras se relaja?						
15. ¿Escucha música al viajar (p.ej. en el coche)?						
16. ¿Escucha música nueva (es decir, música que no ha escuchado antes)?						
17. ¿Asiste a eventos musicales públicos (p.ej. musicales, conciertos o festivales de música)?						
18. ¿Canta, toca algún instrumento musical o silba?						

 © 2025 Giorgos Dritsakis

Parte II

La segunda parte del cuestionario evalúa la importancia que tiene para usted la capacidad de escuchar música, la actitud hacia la música y las actividades musicales descritas en la primera parte del cuestionario. Por favor, conteste a las preguntas marcando una de las siguientes opciones: **1: nada importante; 2: poco importante; 3: algo importante; 4: muy importante; 5: extremadamente importante, N/A: no aplicable**

PERCEPCIÓN MUSICAL	1	2	3	4	5	N/A
1. ¿Qué importancia tiene para usted la capacidad de distinguir distintos ritmos musicales?						
2.¿Qué importancia tiene para usted la capacidad de seguir una melodía musical (es decir, seguir la melodía de una canción o de una pieza musical familiar)?						
3. ¿Qué importancia tiene para usted la capacidad de diferenciar tonos musicales (es decir, agudos y graves)?						
4. ¿Qué importancia tiene para usted la capacidad de reconocer la letra de las canciones?						
5. ¿Qué importancia tiene para usted la capacidad de reconocer los sonidos de diferentes instrumentos musicales?						
6. ¿Qué importancia tiene para usted la capacidad de escuchar el significado de una pieza musical (es decir, la emoción, la razón por la que fue compuesta o el mensaje que intenta transmitir)?						
7. ¿Qué importancia tiene para usted la capacidad de escuchar música sin esfuerzo sin la necesidad de concentrarse?						
8. ¿Qué importancia tiene para usted la capacidad de reconocer una melodía familiar (p.ej. una canción, un/a cantante, una pieza musical)?						
9. ¿Qué importancia tiene para usted la capacidad de valorar la calidad de una interpretación musical (p.ej. cuando alguien canta o toca un instrumento musical)?						
10. ¿Qué importancia tiene para usted tener la sensación de que escucha música igual que otras personas?						
11. ¿Qué importancia tiene para usted escuchar la música afinada?						

DEDICACIÓN A LA MÚSICA	1	2	3	4	5	N/A
12. ¿Qué importancia tiene para usted disfrutar de la música en entornos ruidosos si no hay referencias visuales (p.ej. en una fiesta, en un restaurante o en el coche con el ruido del motor o de la calle de fondo)?						
13. ¿Qué importancia tiene para usted disfrutar de la música en la televisión, en el portátil, en la tablet o en el teléfono?						
14. ¿Qué importancia tiene para usted disfrutar de la música de fondo durante la práctica de actividades como la lectura, la pintura, la jardinería, el ejercicio físico, etc. o mientras se relaja?						
15. ¿Qué importancia tiene para usted escuchar música al viajar (p.ej. en el coche)?						
16. ¿Qué importancia tiene para usted escuchar música nueva (es decir, música que no ha escuchado antes)?						
17. ¿Qué importancia tiene para usted asistir a eventos musicales públicos (p.ej. musicales, conciertos o festivales de música)?						
18. ¿Qué importancia tienen para usted cantar, tocar un instrumento musical o silbar?						

© 2025 Giorgos Dritsakis
